# Fast, facile and thermal damage free nanowelding of Ag nanowire for flexible transparent conductive film by pressure-assisted microwave irradiation

**DOI:** 10.1038/s41598-023-41646-9

**Published:** 2023-09-01

**Authors:** Jong-Min Jeong, Minjeong Sohn, Junghwan Bang, Tae-Ik Lee, Min-Su Kim

**Affiliations:** Advanced Joining and Additive Manufacturing R&D Department, Korea Institute of Industrial Technology, 156, Gaetbeol-ro, Yeonsu-gu, Incheon, 21999 Republic of Korea

**Keywords:** Engineering, Materials science

## Abstract

A fast and straightforward fabrication process for producing a robust, flexible, and transparent conductive film was demonstrated using nanowelding of Ag nanowires through pressure-assisted microwave irradiation. This innovative process effectively reduces the sheet resistance of the Ag nanowire transparent conductive film without causing any thermal distortion to the PET substrate. The microwave irradiation induces nanowelding between Ag nanowires, leading to a decrease in sheet resistance by forming nanowelding junctions. This selective heating of Ag nanowires further enhances the reduction in sheet resistance. Additionally, the application of pressure-assisted microwave irradiation allows the Ag nanowires to be embedded into the PET substrate, resulting in the formation of a robust film capable of withstanding cycling bending stress. The pressure-assisted microwave irradiation process proves to be a strong fabrication method for creating Ag nanowire transparent conductive films, especially when dealing with thermally weak substrate materials.

## Introduction

Flexible transparent conductive films (TCF) have extensive applications in optoelectronics, transparent displays, photovoltaics, and sensors^1^. In order to meet requirements of these applications, the transparent electrodes should possess not only transparency but also electrical conductivity, flexibility, and mechanical reliability. Indium-tin-oxide (ITO) has been a commonly used material for transparent electrodes^2–4^. However, it has some drawbacks when it comes to flexible transparent electrodes, such as its high cost and limitations on the flexibility of electronic devices due to its brittle nature. Therefore, there is a need for alternative materials to replace ITO in flexible electronic applications. Various nanomaterials have been investigated as substitutes for ITO, including carbon nanotubes, graphene, metal nanowires, and others. These alternatives offer potential solutions to overcome the limitations of ITO and pave the way for more flexible and cost-effective transparent conductive films in the field of flexible electronics^5–8^.

Ag nanowires (Ag NW) have been recognized as a promising material for flexible transparent conductive films due to their excellent properties, such as high electrical conductivity, mechanical flexibility, good optical transparency, and low process temperature^9–12^. However, one of the most common intrinsic drawbacks of Ag NW networks is the high sheet resistance between the nanowires^13,14^. To address this issue, numerous research studies have been conducted to lower the contact resistance through various processes, including thermal annealing^15,16^, light treatment^17^, and plasma treatment^18^. In particular, conventional oven treatment has been used to minimize the resistance, but it requires annealing at a high temperature (over 150 ℃) for an extended period. This high temperature exceeds the heat deflection temperature (HDT) of polymeric substrates, leading to thermal distortion of the substrate and subsequently reducing transmittance and flexibility. For instance, the HDT of polyethylene terephthalate (PET) is approximately 65 ℃^19,20^.

Alternatively, the microwave process holds promise as a technology for addressing the high sheet resistance issue of silver nanowire (Ag NW) networks. This process involves the fast and selective absorption of microwaves only by materials like metallic wires and thin films, rather than affecting the polymer substrates based on their electromagnetic characteristics^21,22^. Microwave processes that operate within specific frequency ranges of the electromagnetic spectrum offer several advantages, including shorter process times, lower power consumption, and higher efficiency compared to other conventional methods^23,24^. For instance, Chen et al.^25^ have explored the application of the microwave process in selective absorption through ZnO nanowire (NW)/epoxy composites. They found that ZnO nanowires exhibit excellent electromagnetic wave absorption properties within a specific frequency range. This demonstrates the potential of using the microwave process to address the challenges associated with Ag NW networks and their sheet resistance limitations. Feng et al.^26^ demonstrated the use of the microwave selective sintering effect for carbon nanotubes. Zhao et al.^27^ studied the microwave absorption of Ag NW-filled multi-walled carbon nanotubes (MWCNT) and confirmed the impact of Ag NW on microwave absorption. These microwave processes have shown promise in reducing the sheet resistance of conductive films.

However, the application of Ag NW networks in flexible transparent conductive films presents some challenges. The main issues include defects in surface roughness and weak adhesion between the Ag NW conducting networks and the PET substrate. To address these challenges, researchers have explored additional conducting mediate coatings (such as graphene, polymers, and oxides) to improve the adhesion between the polymer surface and Ag NW, which helps to reduce sheet resistance^28^. Unfortunately, these additional coating processes often come with a trade-off. While they enhance the adhesion and reduce sheet resistance, they can negatively impact the transmittance characteristics of the films. Moreover, these processes usually require complicated multi-step procedures, making them less practical for large-scale production and applications.

**Figure 1:**
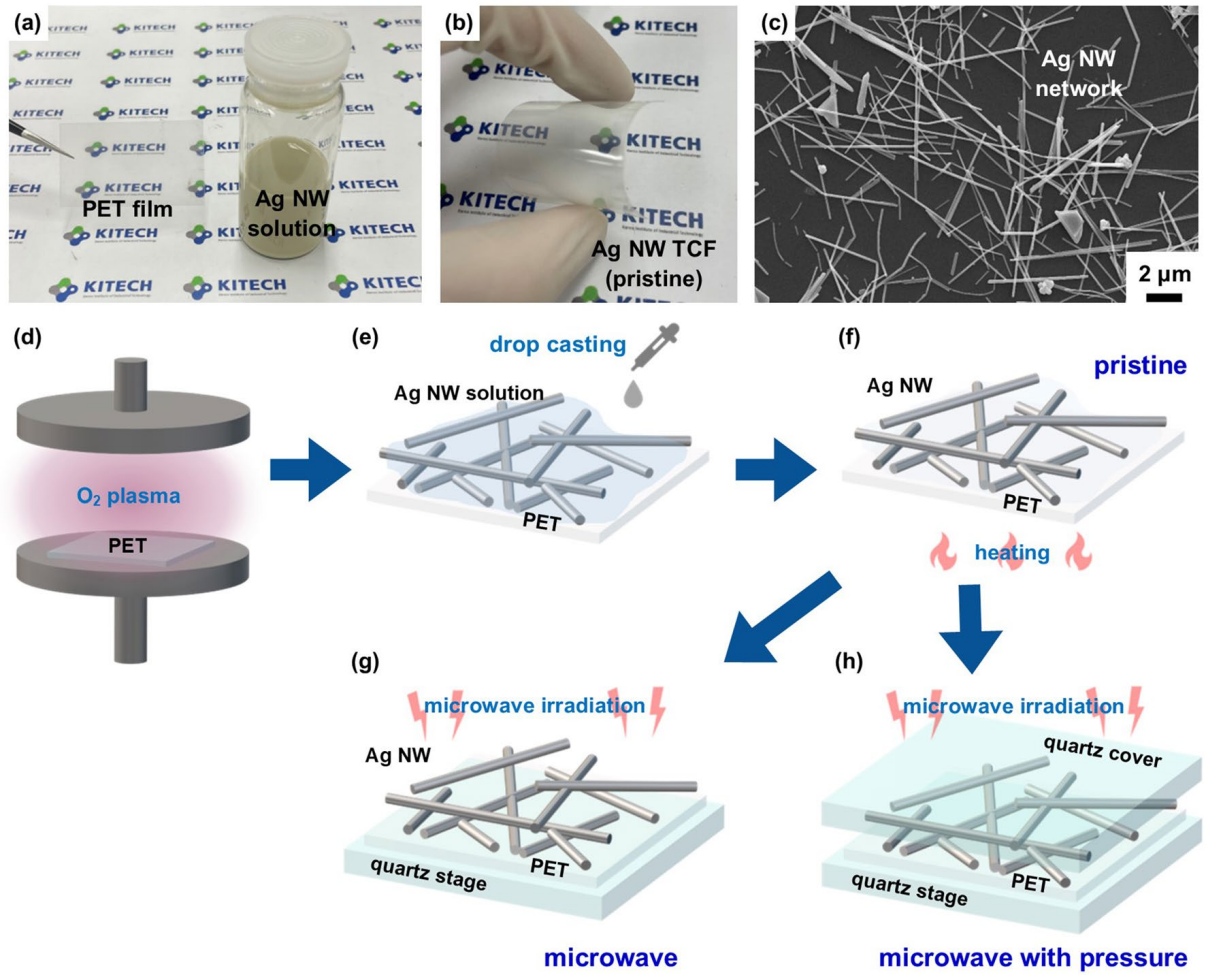
(**a**) PET film and Ag NW solution, (**b**) pristine Ag NW TCF, (**c**) Ag NW morphology for pristine Ag NW TCF. (**d**–**h**) Schematic diagram of fabrication process of Ag NW TCF by microwave nanowelding, (**d**) hydrophilic surface treatment of PET substrate using O_2_ plasma, (**e**) drop casting of Ag NW solution on the PET substrate, (**f**) solvent evaporation from the Ag NW solution in the convection oven, (**g**) microwave irradiation without pressure, and (**h**) microwave irradiation with pressure by quartz cover.

The application of pressure during the microwave process offers a practical solution to achieve low sheet resistance, high transmittance, and improved surface roughness and adhesion of silver nanowire (Ag NW) networks in flexible transparent conductive films. Here, pressure-assisted microwave process for the facile Ag NW transparent conductive film (Ag NW TCF) fabrication method is demonstrated. Ag nanowire transparent PET films was fabricated following sequences: O_2_ plasma treatment, drop casting of Ag NW solution, solvent evaporation and microwave irradiation with and without pressure, as shown in Fig. 1. The transparency and sheet resistance of Ag NW TCF for pristine, microwave, microwave with pressure were investigated.

## Results and discussion

**Figure 2:**
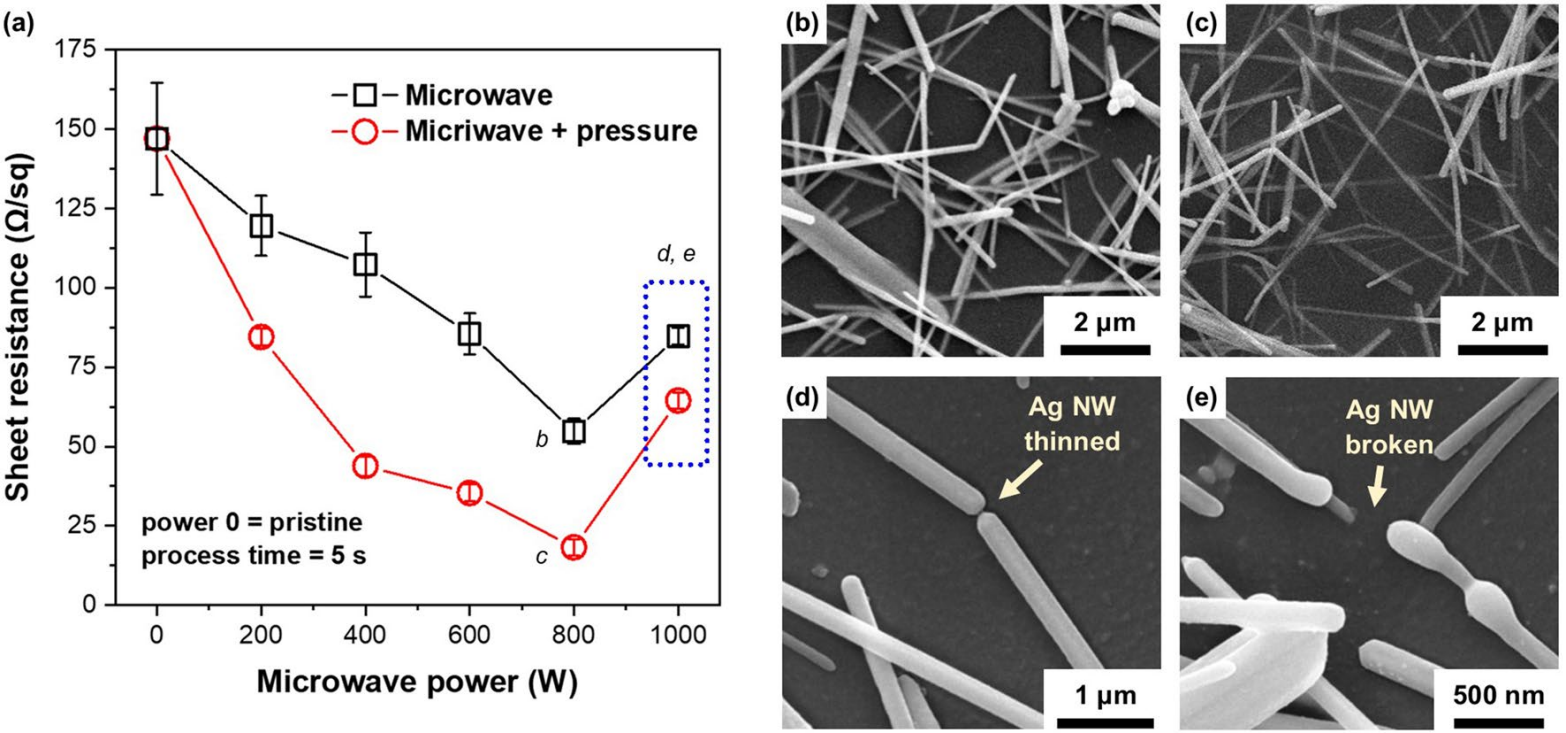
(**a**) Change of sheet resistance of Ag NW TCF as a function of microwave power for microwave and microwave with pressure, where power = 0 means pristine sample, (**b**) Ag NW network formation for microwave with the power of 800 W, (**c**) Ag NW network formation for microwave with the power of 800 W with pressure and (**d**, **e**) morphology of thinned or broken Ag NW after the microwave irradiation with power of 1000 W.

Figure 2a shows the variation in sheet resistance of Ag NW TCF as a function of microwave irradiation power for 5 s. The sheet resistance value for pristine Ag TCF was 147.0 Ω/sq. The trend of the change in sheet resistance with increasing microwave power was the same regardless of whether pressure was applied or not. The microwave sample decreased to 54.7 Ω/sq, and the microwave with pressure sample decreased to 18.2 Ω/sq when 800 W of microwave was irradiated for 5 s on a sample with an initial sheet resistance of 147 Ω/sq. The effect of pressure is also very significant in achieving lower sheet resistance. This is believed to be the result of enhanced physical contact between Ag NWs by the pressure, resulting in a lower resistance value than when only microwave is irradiated. The Ag NW network formation of the TCF irradiated with 800 W microwave for 5 s is shown in Fig. 2b. It appears that the microwave irradiation effectively formed the nanowelding junction between Ag NWs. In the case of the pressure-assisted process, Ag NW embedding into the PET substrate occurred, as shown in Fig. 2c.

In both the microwave and microwave with pressure cases, the sheet resistance increased when the irradiation power of microwave increased from 800 to 1000 W. Through SEM observation, partial thinning or breakage of Ag NW has been confirmed, as shown in Fig. 2d,e. This is believed to be the result of microwave irradiation exceeding the optimal output conditions, which leads to excessive heat generation and fragmentation due to Ag diffusion. This phenomenon can be explained by the Reyleigh instability of nanowire^29^. When metal NWs are subjected to energy such as heat or laser, surface diffusion occurred to reduce their surface area, causing them to powder and shatter. More than 1000 W of microwave irradiation appears to cause excessive heating of Ag NWs.

**Figure 3:**
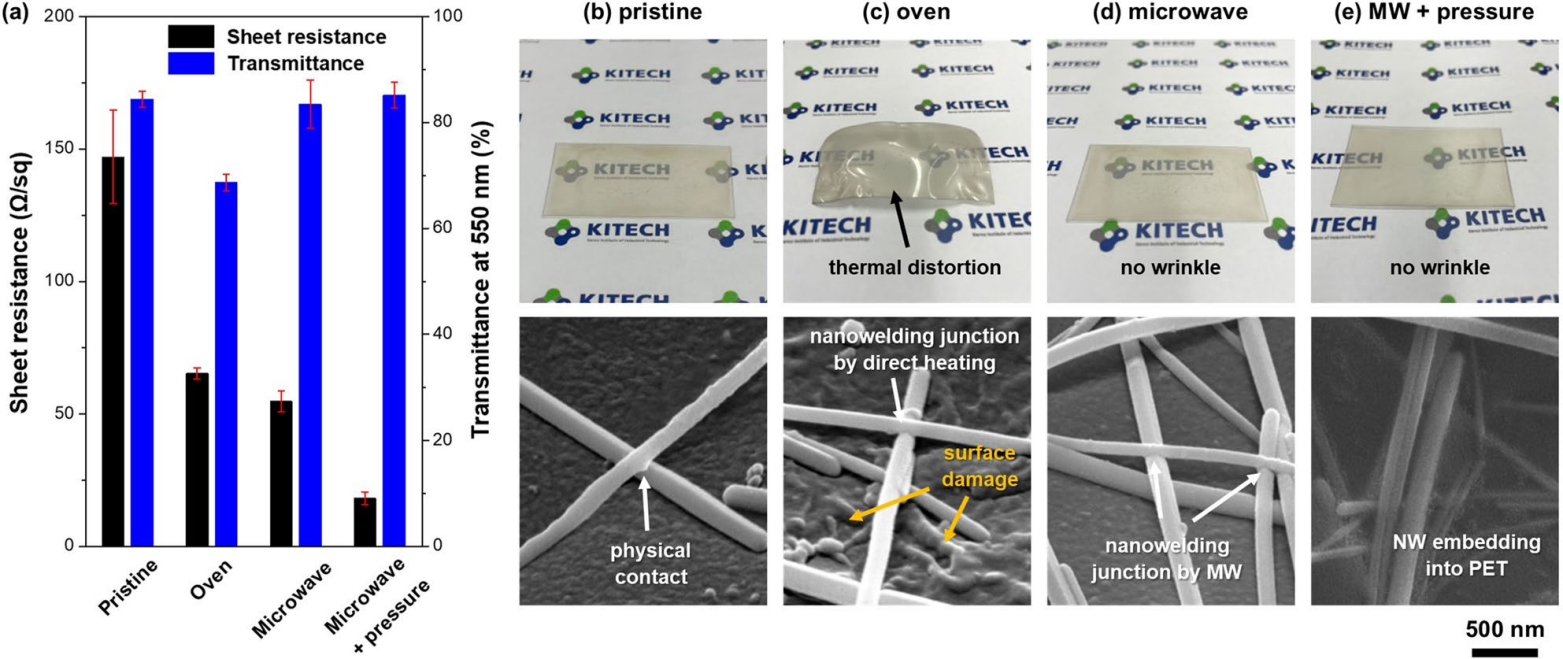
(**a**) Sheet resistance and transmittance at 550 nm of Ag NW TCF for pristine, oven, microwave and microwave with pressure. Appearance of Ag TCF and SEM images on the Ag NW network formation for (**b**) pristine, (**c**) oven (150 °C, 20 min), (**d**) microwave (800 W, 5 s) and (**e**) microwave with pressure (800 W, 5 s).

Figure 3 exhibits comparative results of sheet resistance and transparency and morphologies of Ag NW TCFs for pristine and fabricated using oven, microwave, and microwave with pressure. In the case of a pristine sample, it exhibits a relatively high sheet resistance because the electrically conductive pass was formed through the physical contact between Ag NWs only (Fig. 3b). When the sample was heated in an oven at 150 °C for 20 min, the sheet resistance decreased compared to the pristine sample due to surface diffusion caused by heat, resulting in the formation of nanowelding junctions. However, a severe thermal distortion of the PET substrate was observed (Fig. 3c), leading to a decrease in transmittance of 69%. In cases of microwave irradiation, there are no substrate wrinkles, unlike those made by the oven process. As a result, the transmittances of the microwave sample and microwave with pressure sample were almost similar to that of the pristine sample. The nanowelding junctions between Ag NWs were formed by microwave radiation (Fig. 3d), resulting in low sheet resistance, and NW embedding into PET substrate occurred when pressure was applied (Fig. 3e). From these results, it is possible to lower the sheet resistance by microwave irradiation without any thermal damage to the substrate.

In the case of conductive materials, the heating mechanism generated by microwave-material interaction is a conduction loss. In the case of non-magnetic materials, only the electric field component of the microwave has an effect on them. The external electric field induces electric field oscillation inside the non-magnetic metallic materials, resulting in the generation of uniform volumetric heating^30^. Metallic materials are commonly regarded as opaque in the microwave with negligible energy absorption because of their limited skin depth compared with their volume^30^. The skin depth, *δ*, is the ability of electromagnetic radiation to penetrate into the material body, which can be calculated by Eq. (1):

1 $$\delta = \frac{1}{{\sqrt {\pi f\mu \sigma } }}$$

where *f*,* μ*, and *σ* are the frequency of the excitation signal, magnetic permeability, and electrical conductivity, respectively^31^. For silver, *μ* = 4π × 10^7^ H/m, *σ* = 6.09 × 10^7^ Ω^-1^ m^-1^, so skin depth, *δ*, is 1.28 μm at a frequency of 2.45 GHz. In the case of bulk Ag, it is not possible to heat Ag body by microwave due to the limited skin depth, whereas for Ag NW, it is possible to achieve uniform heating because the diameter of Ag NW is less than the skin depth of Ag.

**Figure 4:**
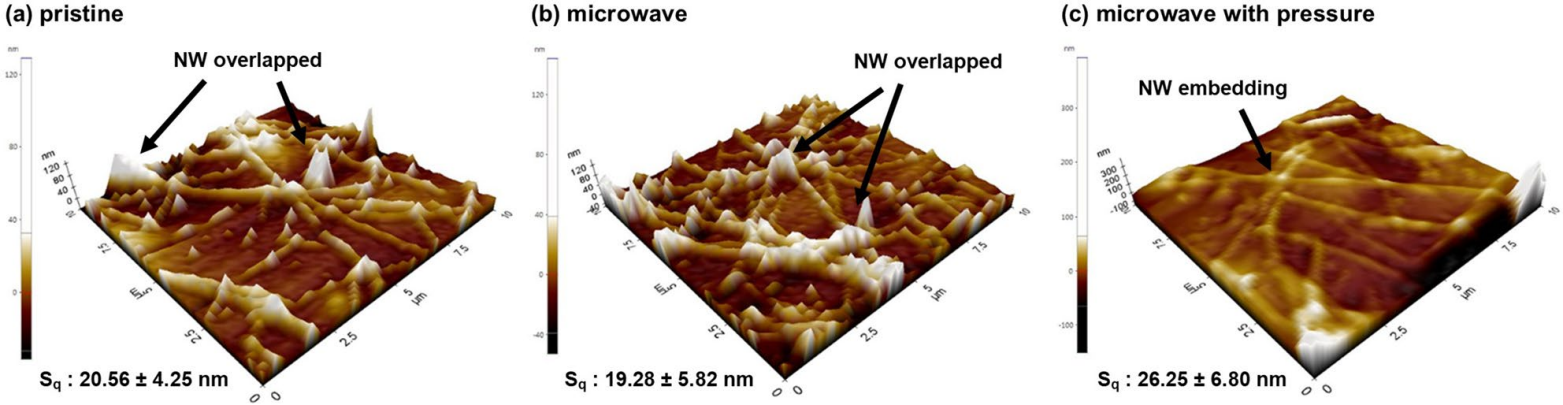
Topographical images and root mean square height (S_q_) values for (**a**) pristine, (**c**) microwave (800W, 5 s), and (**c**) microwave with pressure (800W, 5 s).

Figure 4 exhibits topographical images of Ag NW TCF surfaces for the pristine, microwave, and microwave with pressure samples. Based on the measurement results, the root mean square roughness (S_q_) for pristine, microwave, and microwave with pressure samples are 20.56 ± 4.25 nm, 19.28 ± 5.52 nm, and 26.25 ± 6.80 nm, respectively. The data shows that the surface roughness of the Ag NW TCF fabricated by microwave irradiation with pressure slightly increases after the embedment of Ag NW into the PET substrate. However, this increase is not significant. This indicates that the pressure-assisted microwave process, which allows the Ag NW conducting networks to be embedded into the substrate, has a limited impact on the overall surface roughness.

**Figure 5:**
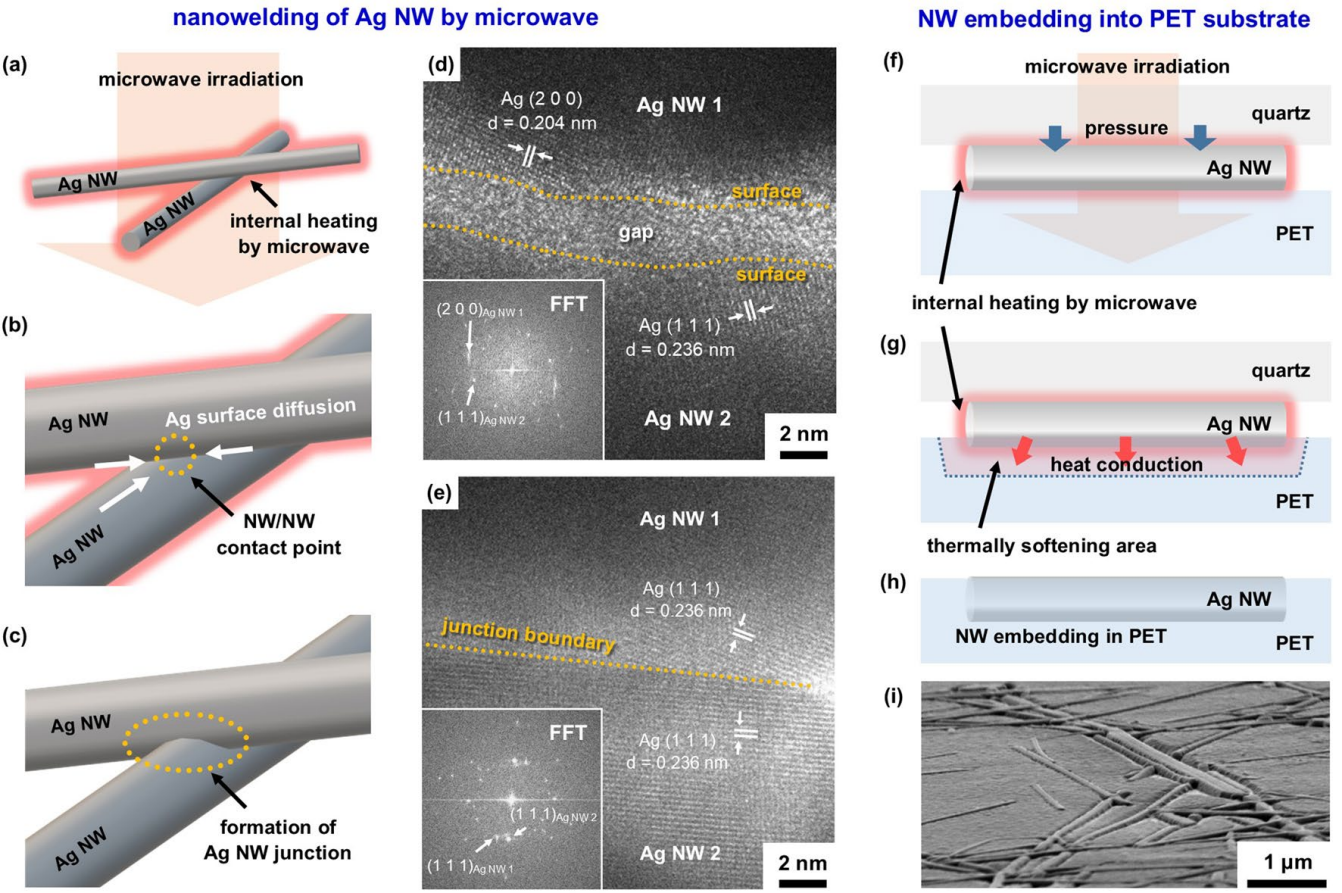
(**a**–**e**) Schematic diagram of possible mechanism of nano-welding between two Ag NWs: (**a**) internal heating of Ag NW by microwave irradiation, (**b**) Ag surface diffusion to NW/NW contact point and (**c**) formation of Ag NWs junction. High-resolution TEM (HRTEM) lattice images of Ag NW junction for (**d**) pristine Ag NW TCF and (**e**) Ag NW TCF formed by microwave irradiation with the power of 800 W for 5 s. (**f**–**i**) Schematic diagram of possible mechanism of Ag NW embedding into PET substrate by microwave irradiation and applying pressure: (**f**) internal heating of Ag NW by microwave irradiation, (**g**) local thermal softening behavior of PET substrate by the heat conduction from Ag NW and (**h**) Ag NW embedding into PET substrate. (**i**) tilted SEM image of embedded Ag NW.

In Fig. 5, we describe mechanisms of formation for the robust Ag NW TCF by pressure-assisted microwave irradiation. Figure 5a–c exhibits a possible mechanism of Ag NW junction formation by microwave irradiation. The microwave irradiation induced internal heating of individual Ag NW, as shown in Fig. 5a with the increase of temperature, the surface atoms of Ag are located in an unstable state and migrate in order to reduce surface area. When the contact point of Ag NWs existed, Ag atoms migrated at the contact point and then formed junctions between two Ag NWs, as shown in Fig. 5b. As results, Ag NW junction with the metallic interconnection was formed by the microwave irradiation, reducing the sheet resistance of the entire TCF.

Figure 5d,e exhibits direct evidence for the formation of the Ag NW junction by microwave irradiation. In the case of pristine Ag NW TCF, there is a narrow gap between two different Ag NWs. The gap is a capping layer on the surface of Ag NW. Because of non-direct contact between two Ag NWs, the electrical interconnection was limited. After the microwave irradiation, it is clearly confirmed that two Ag NWs were connected to each other from the metallic lattice image using TEM, as shown in Fig. 5e. There is no void at the junction boundary between two Ag NWs, which is why the electrical resistance decreased by the microwave irradiation.

The applied pressure during the microwave irradiation process can play a role in reducing the sheet resistance of Ag NW TCF and enhancing Ag NW adhesion to the PET substrate. The applied pressure through the quartz pressure tool causes the Ag NW and PET to contact more closely, as shown in Fig. 5f. As previously explained, microwave irradiation causes internal heating of the Ag NW, and this heat is transferred to the PET substrate through heat conduction, causing softening of the PET substrate to occur at the point of contact with the Ag NW, as shown in Fig. 5g. The continuous pressure applied during the MW process leads Ag NW to penetrate into the PET, as shown in Fig. 5f. The tilted SEM images of Ag NW TCF fabricated microwave irradiation with pressure, as shown in Fig. 5i, clearly exhibited the Ag NW embedding into PET substrate.

**Figure 6:**
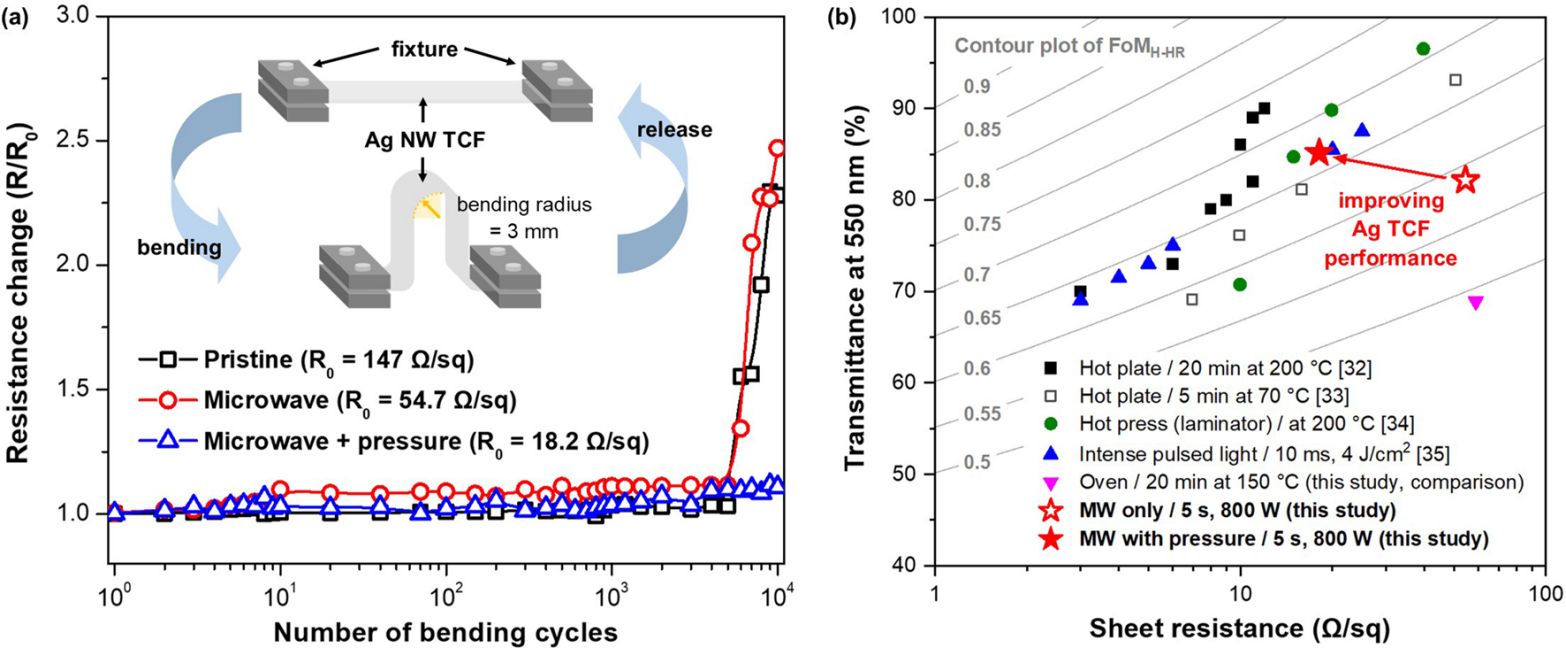
(**a**) Sheet resistance changes as a function of bending cycles up to 10,000 cycles for pristine Ag NW TCF, and fabricated Ag NW TCFs using microwave irradiation or microwave irradiation with pressure, and (**b**) comparison of transmittance (T) at 550 nm and sheet resistance (R_s_) of the Ag nanowire transparent conductive films fabricated by various processes. Reference data obtained using only Ag nanowires without any other additives were selected. Inset contour plots indicates figure of merit (FoM) values from 0.5 to 0.9 calculated by FoM Haacke high resolution (FoM_H-HR_).

Figure 6a exhibits resistance change during the cyclic bending for the pristine, microwave sample and microwave with pressure sample. In the case of pristine and microwave samples, the resistance changes rapidly increased after 5000 cycles, and it showed a resistance increase of more than twice the initial sheet resistance value after 10,000 cycles. In contrast, the microwave with a pressure sample showed almost no resistance change up to 10,000 cycles. This effect is due to the Ag NW embedding into the PET substrate, as shown in Fig. 3e, where the Ag NWs are well attached to the substrate without separating from it even when bending occurs.

Figure 6b exhibits the comparison results of the performances of Ag NW transparent conductive film by the various fabrication methods in the previous reports. The fabrication processes for comparison include hot plate^32,33^, hot press^34^, and intense pulsed light (IPL)^35^. For a precise comparison, the reference data in Fig. 6b were extracted from the performances of Ag NW transparent conductive films using only Ag NW without any additives for enhancing electrical conductivity. An inset contour plot indicates a figure of merit (FoM), which is an evaluation method for the quality of transparent conductive film with two important parameters, such as transmittance and sheet resistance^36^. Here, we adopted the FoM Haacke high resolution method, called FoM_H-HR_^37^, which is calculated by the following equation:

2 $$FoM_{H - HR} = \frac{T}{{\sqrt[{12}]{{R_{s} }}}},$$

where *T* and *R*_*s*_ are the transmittance and sheet resistance of transparent conductive film, respectively. With a FoM value close to 1, it is possible to conclude that the performance of transparent conductive film is superior.

Among these, transparent conductive film produced with Ag NW by hot plate^32,33^, and hot pressing^34^ methods, which are direct heat transfer methods, exhibit well FoM performance around 0.7 compared to other technologies. However, direct heat transfer methods are not suitable for thermally sensitive substrates because they can cause thermal distortion when applied to materials. The IPL method is also a very powerful fabrication method for transparent conductive film, which exhibits a high FoM value with 0.63–0.67^35^. Microwave irradiation with pressure also shows good performance, with the FoM value of 0.669. In the case of pristine Ag NW TCF, the FoM value was 0.557. After microwave irradiation, the FoM value slightly increased to 0.588.

## Conclusion

In summary, the microwave irradiation process is effective in forming nanowelding junctions between Ag NWs without causing any thermal damage to the substrate, thereby reducing the sheet resistance of the conductive film. The application of pressure during the microwave process enhances the physical contact between Ag NWs, leading to the formation of more conductive paths and further lowering the sheet resistance compared to microwave radiation alone. The pressure-assisted microwave process enables the easy embedding of Ag NW conducting networks into the substrate, resulting in improved stability under bending deformation. This technique facilitates the fabrication of Ag NW transparent conductive films with desirable properties, such as low sheet resistance, high transmittance, and enhanced mechanical reliability, making it a promising method for flexible electronic applications.

## Methods

### Materials

A flexible transparent PET substrate with a thickness of 78 μm was utilized. As the conducting material, a commercially available polyvinylpyrrolidone-stabilized Ag NW solution in ethanol was employed, with a concentration of 5 mg/mL. The Ag NWs had a diameter of 100 nm and lengths ranging from 5 to 50 μm. Prior to application, the Ag NW solution was diluted to a concentration of 0.3 mg/mL. The appearance of PET film and Ag NW solution can be found in Fig. 1a–c.

### Nanowelding process by pressure-assisted microwave irradiation

The schematic illustration of the fabrication process using microwave irradiation of Ag NW transparent conductive film is shown in Fig. 1d–h. The PET film with an area of 5 cm × 5 cm was cleaned with ultrasonic cleaner within ethanol to remove surface contamination, and then treated with O_2_ plasma to enhance the wettability of the Ag NW solution. The O_2_ plasma surface treatment can enhance the surface hydrophilicity, resulting in a fast and uniform spread of Ag NW solution on the substrate^38^. Then, the Ag NW solution was coated on to the PET film by drop-casting, as shown in Fig. 1e. Ag NW solution coated PET film was dried in the convection oven at 60 °C for 10 min to evaporate the solvent, which was named the pristine (Fig. 1f). After that, 2.45 GHz microwave was irradiated to the pristine Ag TCF with and without applying pressure for the verification of the effects of microwave and pressure. The pressure was applied by the weight of the quartz cover, where the converted pressure was 2.3 kPa. The microwave power varied from 200 to 1000 W, and the irradiation time was 5 s.

### Characterization

The sheet resistances and transmittances of the Ag NW TCFs were measured using four-point probe system (SR2000N, AIT) and UV/Vis/NIR spectrophotometer (LAMBDA 750, Perkin Elmer), respectively. The morphologies of the Ag NW TCF were obtained by scanning electron microscopy (SEM, Quanta 200F, FEI). The formation of nanowelding junctions was observed using transmission electron microscopy (TEM, JEM-2100F, JEOL). A cyclic bending test was performed by a bending tester to confirm the electrical–mechanical stability characteristics. Surface topography was measured using atomic force microscopy (AFM, XE-100, PSIA).

## Data Availability

The data is available upon reasonable request to the corresponding authors.
